# Supplementary material for: NGS Sequencing Reveals New UCP1 Gene Variants Potentially Associated with MetS and/or T2DM Risk in the Polish Population—A Preliminary Study
Source: Genes (Basel). 2023 Mar 24;14(4):789. doi: 10.3390/genes14040789 (PMC10137642; doi:10.3390/genes14040789)
Supplement: Supplementary file 1 [file genes-14-00789-s001.zip › Figure S1.pdf]

Supplementary Materials

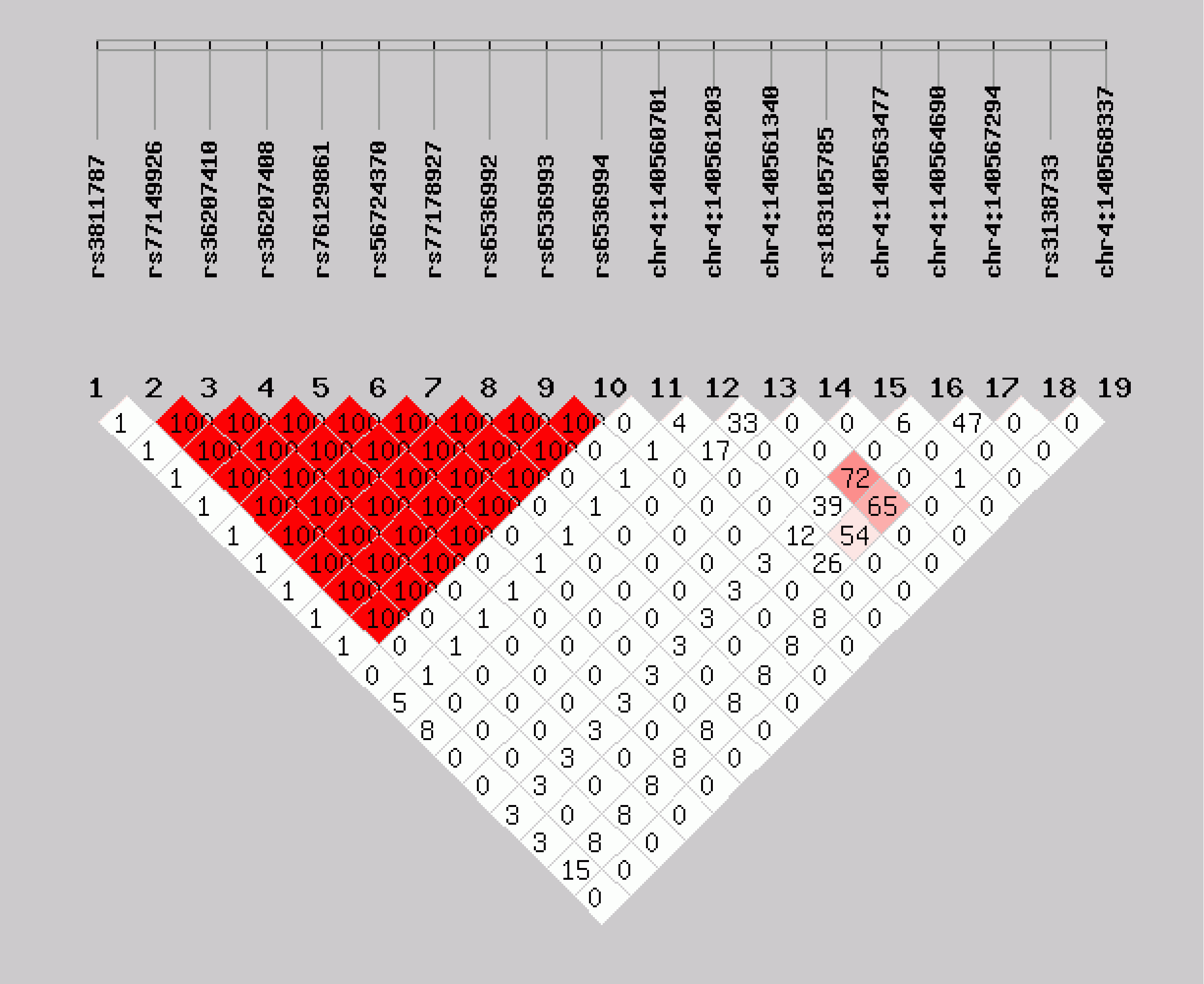

Figure S1 Results of the LD analysis with the use of SHEsis software on the genotyping data from the control group for SNVs potentially associated with MetS or MetS with T2DM). Red color indicates SNVs in strong LD and pink color indicates SNVs in moderate LD. Presented values were calculated using Shesis software (available at http://analysis.bio-x.cn/myAnalysis.php).
